# *Methylobacterium* Species Promoting Rice and Barley Growth and Interaction Specificity Revealed with Whole-Cell Matrix-Assisted Laser Desorption / Ionization-Time-of-Flight Mass Spectrometry (MALDI-TOF/MS) Analysis

**DOI:** 10.1371/journal.pone.0129509

**Published:** 2015-06-08

**Authors:** Akio Tani, Nurettin Sahin, Yoshiko Fujitani, Akiko Kato, Kazuhiro Sato, Kazuhide Kimbara

**Affiliations:** 1 Institute of Plant Science and Resources, Okayama University, Okayama, Japan; 2 Egitim Fakultesi, Mugla Sitki Kocman University, Mugla, Turkey; 3 Faculty of Engineering, Shizuoka University, Hamamatsu, Japan; Colorado State University, UNITED STATES

## Abstract

*Methylobacterium* species frequently inhabit plant surfaces and are able to utilize the methanol emitted from plants as carbon and energy sources. As some of the *Methylobacterium* species are known to promote plant growth, significant attention has been paid to the mechanism of growth promotion and the specificity of plant–microbe interactions. By screening our *Methylobacterium* isolate collection for the high growth promotion effect *in vitro*, we selected some candidates for field and pot growth tests for rice and barley, respectively. We found that inoculation resulted in better ripening of rice seeds, and increased the size of barley grains but not the total yield. In addition, using whole-cell matrix-assister laser desorption/ionization- time-of-flight mass spectrometry (MALDI-TOF/MS) analysis, we identified and classified *Methylobacterium* isolates from *Methylobacterium*-inoculated rice plants. The inoculated species could not be recovered from the rice plants, and in some cases, the *Methylobacterium* community structure was affected by the inoculation, but not with predomination of the inoculated species. The isolates from non-inoculated barley of various cultivars grown in the same field fell into just two species. These results suggest that there is a strong selection pressure at the species level of *Methylobacterium* residing on a given plant species, and that selection of appropriate species that can persist on the plant is important to achieve growth promotion.

## Introduction

Various kinds of microorganisms interact with plants, and their presence unequivocally affects plant growth. Microorganisms associated with plants include epiphytes and endophytes. The former may inhabit the rhizosphere or phyllosphere, whereas the latter inhabits within plant tissues without causing apparent disease symptoms. Understanding the ecology, roles, and function of these microorganisms is crucial for effective agriculture to support food production. Many bacterial species have been isolated and tested for their ability to promote plant growth, they are called plant-growth-promoting bacteria or rhizobacteria (PGPB or PGPR, respectively) [1].

Metagenomic analysis revealed that leaf epiphytes are dominantly composed of *Alphaproteobacteria*, including *Methylobacterium* and *Sphingomonas* as the major genera [2]. *Methylobacterium* species benefit from the methanol produced by plants as a product of pectin demethylation [3]. It is believed that the wide distribution and predomination of the genus on the plant surface is due to the methylotrophy of the genus. The abundance of *Methylobacterium* in different plant species may vary markedly depending on the plant species, site, and growth stage [4,5]. Culture-independent community composition studies determined that *Methylobacterium* spp. and other methylotrophs are consistently part of the phyllosphere microbiota of various plant species [6]. In previous ecological and taxonomical studies, the plant–*Methylobacterium* association with rice and barley plants has also been reported [7–10].

*Methylobacterium* species are capable of promoting plant growth or seedling germination through the production of ACC (1-aminocyclopropane 1-carboxylate) deaminase [11], indole acetic acid [12], cytokinin [8,11,13,14], acids to release insoluble phosphate [15], and siderophore [16]. Some species are able to fix nitrogen [17] and antagonize pathogens [18]. However, it remains poorly understood which of and how much the aforementioned traits contribute to the effect. Considering their abundance and growth promotion effect, research on the interaction between *Methylobacterium* and plants is important, especially in terms of utilizing bacteria for agricultural purposes.

In our previous study [19], we isolated approximately 200 *Methylobacterium* isolates from various plant species, and classified them with a method utilizing whole-cell matrix-assisted laser desorption ionization time-of-flight mass spectrometry (WC-MS) analysis. In this method, the microbial cells are directly used as samples for mass spectrometry, and protein peak patterns detected within m/z 2000 to 20,000 are utilized as fingerprints. Though differences in the cultivation media affect the spectra, since most of the detected peaks can be attributed to ribosomal proteins, essentially the same peaks with different intensities could be observed. Our results showed that peak pattern did not change during the two-week cultivation time. The cluster analysis using the spectra data can be used to rapidly identify and classify the unidentified bacteria without sequencing marker genes like the 16S rRNA gene. Several hundred samples can be processed within an hour. In the study, we generated a spectra database of almost all *Methylobacterium* type strains, and examined the relationship between their spectra similarity and pairwise 16S rRNA gene identity. The spectra comparison could differentiate species more efficiently than sequencing. Whereas the threshold for species discrimination is 97% [20] or 98.6% [21] for 16S rRNA gene sequences identity, a spectral similarity of 0.5 (from 0 to the maximum 1.0 based on our manual calculation) could serve as a threshold for species discrimination, even for close species sharing more than 98.6% 16S rRNA gene identity. Bruker Daltonics provides BioTyper software that automates similarity calculations, the creation of dendrograms, and the identification of unidentified microorganisms by comparing the spectra with a spectra library. The commercialized library contains a limited number of environmental microorganism spectra. Recently, the technique has been increasingly used to identify microorganisms at the species level (and in some cases at the strain or subspecies level) [22].

As a result of our previous study, we found that isolates belonging to some specific species were those of the most frequently isolated species from various plants as generalists, and many of those from bryophytes did not belong to known species. Thus, there seemed to be a specificity of interaction at the species—species level between bacteria and plants, which has also been demonstrated by culture-independent studies [23]. The factors that regulate the specificity are largely unknown. It is also unknown whether the plants can tolerate the presence of *Methylobacterium* species that is not their specific counterpart. These points are also crucial to understand in order to utilize *Methylobacterium* species for agricultural purposes.

In the present study, we aimed to identify the most potent strains capable of promoting rice and barley growth from our *Methylobacterium* library, and tested them in practical field and greenhouse pot experiments. In addition, we isolated and identified *Methylobacterium* species from these plants to estimate the interaction specificity between the bacteria and plants, using WC-MS analysis.

## Materials and Methods

### Screening of growth promoting effect of *Methylobacterium* for rice and barley seedlings

*Oryza sativa* cultivar Nipponbare seeds were husked, and sterilized by passage through 70% ethanol for 3 min, 3% sodium hypochlorite containing 0.02% Tween 20 for 30 min, and sterile water 5 times. Twenty seeds were placed with their embryo underneath on 50 ml of solidified 0.8% agar (TC-6, Ina Food Industry Co.) prepared on a square plate (144 mm × 104 mm × 16 mm). Since the inoculants might confer a growth promotion effect under a stressed condition, an agar plate containing 50 mM NaCl was also used. Microbial cells grown in 5 ml methanol medium [24] for 4 to 5 days were washed with sterile water, and suspended in 5 ml water. The cell suspension (OD_600_ = 1.0) was applied to the rice seeds (5 μl/seed). The seeds were then incubated at 4°C for 2 d, and allowed to grow at 25°C (8 h light/16 h dark cycle) for 10 days. The plates were tilted to allow the plant roots to grow downwards on the agar surface. The fresh weight and the length of roots and shoots were recorded.

Seeds of the *Hordeum vulgare* cultivar Akashinriki were sterilized with the same procedure as that used for rice, described above. Seven seeds were put on a sterile soil extract solidified with 0.8% agar (40 ml) prepared in a transparent plastic box (6 cm × 6 cm × 10 cm). The soil extract was prepared with 400 g of field soil and 2.0 l of water. The soil suspension was autoclaved (121°C for 40 min) and centrifuged (3,000 rpm, 5 min) to remove soil, and the supernatant was used as a soil extract. Five microliters of microbial cell suspension (OD_600_ = 1.0) were applied to each seed. The boxes were incubated at 4°C in the dark for 2 days and then at 23°C in an 8 h/16 h light cycle for 25 days. The seedling growth was evaluated by measuring the shoot and root length, and fresh weight.

### Field experiment of rice cultivation

*Oryza sativa* cultivar Nipponbare seeds were husked and sterilized as described above, and placed on a filter paper (25 seeds/paper, 9 cm diameter). Five milliliters of washed microbial cell suspension (OD_600_ = 1.0) of the selected strains were added to the paper, and the seeds were allowed to germinate for 4 d under the same condition as above. The seedlings were then transferred onto a plastic mesh floated on Kimura B medium (24 mg [NH_4_] _2_SO_4_, 67 mg MgSO_4_·7H_2_O, 9 mg KNO_3_, 42 mg Ca[NO_3_] _2_·4H_2_O, 12 mg KH_2_PO4, 8 mg NaEDTA-Fe·3H_2_O, 1 mg MnCl_2_·4H_2_O, 0.6 mg H_3_BO_3_, 20 μg [NH_4_] _6_Mo_7_O_24_·H_2_O, 4 μg ZnSO_4_·7H_2_O, and 4 μg CuSO_4_·5H_2_O per liter). After 2 weeks, the seedlings were transplanted into a paddy field at the Institute of Plant Science and Resources (IPSR) at Okayama University in 2010 (10 plants/lane, 4 lanes for each treatment; the lanes were randomized). Fertilizer was not applied. After 4 months, the inoculation effect was evaluated as the means of measuring shoot dry weight, number and weight of panicles, grains, and ripened grains. Grains that sank in water of density 1.06 (60 g/l NaCl) were regarded as ripened grains.

### Pot experiment of barley cultivation

Sixteen *Hordeum vulgare* cultivar Akashinriki seeds received 8 ml in each bacterial suspension (OD_600_ = 1) on a filter paper placed on a plastic plate (diameter 9 cm), and were incubated at 22°C for 4 days in the dark. Four seedlings were transplanted onto soil prepared in an unglazed pot (14 to 22 [bottom to top] cm diameter and 17 cm height). Four replicates were prepared for each treatment, and the plants were allowed to grow in a greenhouse, receiving appropriate watering. After 4.5 months, the effect of inoculation was evaluated by measuring plant height, dry weight, grain number and weight, and protein and starch content. Grain size was measured using random 80 grains from the mixed pool of treated plants.

### Protein and starch content analysis

The protein content of the husked seeds was analyzed in duplicate using the Kjeldahl method with 100 g grains of the mixed pool of treated plants. The starch content was analyzed in triplicate using a Megazyme total starch assay kit.

### Recovery and identification of *Methylobacterium* species from leaves of inoculated rice seed

At the harvest, the rice leaves were sampled and subjected to *Methylobacterium* species isolation. We pooled young leaves from plants of the same treatment (one whole leaf from an individual plant: total 20 leaves). The leaf samples were put in sterile 0.85% NaCl, and vortexed vigorously for 30 s. The resultant suspension was spread onto solidified methanol medium [24] containing 50 mg/l cycloheximide. The pink colonies appeared after 3 to 4 days at 28°C and were isolated and purified by streaking on plate media of the same composition. Twenty isolates were obtained from each treatment. All isolates were subjected to WC-MS analysis as reported previously [19]. Since cultivation time and media might affect the spectra, spectra data were obtained using 3- to 4-d-old culture on solid methanol media. For WC-MS clustering, a main spectra projection (MSP) dendrogram was constructed with MALDI Biotyper 3.0 (Bruker Daltonics) in the default setting. MSP creation settings were maximum mass error of each single spectrum, 2000; desired mass error for the MSP, 200; desired peak frequency minimum (%), 25; maximum desired peak number for the MSP, 70; and signal to noise ratio, 3. MSP dendrogram creation settings were distance measure, correlation; linkage, average; score threshold value for a single organism, 300; and score threshold value for related organisms, 0. The *Methylobacterium* type strains obtained from culture collections were included in the analysis. Representative isolates in the MSP dendrogram were subjected to 16S rRNA gene sequencing and phylogenetic analysis, as reported previously [19]. Identification of phylogenetic neighbors and the calculation of pairwise 16S rRNA gene sequence similarities were achieved using the EzTaxon-e server [25].

### Identification of *Methylobacterium* isolates from various barley and wheat cultivars

In 2010, *Methylobacterium* isolates were obtained from leaf samples of 47 barley cultivars and one wheat cultivar (S1 Table) grown in the field at the IPSR, using cycloheximide-containing methanol medium. These cultivars were collected from different areas of the world and represent the key haplotypes currently used in the barley research community. Three isolates from each cultivar were subjected to WC-MS analysis, followed by 16S rRNA gene sequencing for representatives from the clusters as described above. All necessary permits from Field Director of IPSR (Dr. Masahiko Maekawa) were obtained for the described field studies.

### Nucleotide accession numbers

The 16S rRNA gene sequences determined in this study have been submitted to the DDBJ database under the accession numbers AB986540-AB986553.

### Statistical analysis

The statistical significance of the inoculation effect was evaluated by analysis of variance (ANOVA) followed by Dunnett’s test or Fisher’s least significant difference test to determine *p* values for individual groups: using statistical software GraphPad Prism 6 was utilized.

## Results and Discussion

### Screening of *Methylobacterium* species for high rice growth promotion ability

Among the approximately 200 *Methylobacterium* isolates that we obtained in our previous studies [19,24], a sub-library of 77 isolates was selected based on their identity by WC-MS analysis (S2 Table) and subjected to screening. The library contains five eukaryotes and nine bacteria other than *Methylobacterium* species. They were selected based on their positions in the WC-MS dendrogram. They are considered to be unique to each other, and their 16S rRNA genes (or internal transcribed spacer [ITS] region) have been sequenced.

Since it was difficult to test 77 isolates at a time, we separated them into 7 groups, and prepared a control each time. The control experiments under the normal condition and the 50 mM NaCl condition showed shoot length of 5.30 ± 0.22 cm and 3.46 ± 0.21 cm, root length of 8.7 ± 0.37 cm and 7.30 ± 0.35 cm, and fresh weight of 0.060 ± 0.003 g and 0.052 ± 0.002 g, respectively (shown as mean ± standard error of the mean, n = 17 control experiments). The *p* values for the effect of NaCl on the control experiments were <0.0001 for shoot length, 0.008 for root length, and 0.014 for fresh weight (n = 17, Student’s *t*-test), suggesting that the 50 mM NaCl was indeed a stress for rice seedlings.

Through the first screening (S2 Table), the isolates that showed a growth promotion effect were chosen for the second screening under the same condition. Among them, isolates 66g and 92a that showed a reproducible growth-promoting effect under the normal condition and in the presence of 50 mM NaCl, respectively, were selected for field experiments. In addition, *M*. *aquaticum* strain 22A and *M*. *gnaphalii* strain 23e^T^ [26] were also selected for field experiments due to their potential to enhance the growth of various plants [24] and the high ability to produce PQQ [19,24,27], respectively, although they did not show a growth promotion effect in the screening. It is of note that we did not observe any correlation between PQQ productivity of the cells cultured in vitro [19] and the rice seedling growth in this study (data not shown).

### Results of field rice cultivation

The results of the field experiments are summarized in Table 1. The inoculation of isolates 66g and 92a resulted in a lower yield of total grains (grain weight per plant, 87.7% and 86.5%, respectively, compared to the non-inoculated control) and that of ripened grains (93.3 and 88.6%, respectively). The weight of ripened 100-grains was, however, increased compared to that of the control experiment. These results suggested that the individual size of ripened grains was improved. Thus, the inoculation did not result in a greater yield but resulted in an increased size of ripened grains. The protein content tended to decrease and the starch content tended to increase in the treated rice. The biological mechanism underlying these effects is unknown; however, the decreased number of grains as the reduced availability of sink organs for fixed carbon can be considered the main cause for the increased seed size. It is reported that cytokinin controls seed number and size [28,29], and that *Methylobacterium* species are able to synthesize trans-zeatin [8,14]. The effect of *M*. *aquaticum* strain 22A and *M*. *gnaphalii* strain 23e^T^ inoculation did not show a statistically significant difference, but a similar tendency was observed for increased ripened 100-grains weight and starch content.

**Table 1:** Growth of *Methylobacterium*-inoculated rice.

Inoculated strains	Shoot dry weight (g per plant)	Panicles (no. per plant)	Panicle weight (g per plant)	Grains weight (g per plant)	Grains (no. per plant)	100-grain weight (g)	Ripened grains weight (g per plant)	Ripened grains (no. per plant)	Ripened 100-grains weight (g per plant)	Ripened grains (%)	Ripened grains per panicle (no.)	Protein content (%)	Starch content (%)
control	75.3±20.9	23.9±4.4	39.4±7.5	37.4±7.0	1730±378	2.13±0.12	30.4±6.1	1230±245	2.46±0.06	71.2±4.9	51.6±5.1	7.4	68.5±2.7
66g	68.8±21.4	19.9±5.5**	34.8±9.0	33.0±8.5	1460±440*	2.25±0.13	28.4±7.6	1130±297	2.52±0.05***	76.9±4.4***	56.6±6.2*	7.1	70.1±0.4
92a	73.5±22.3	21.4±6.3	34.5±9.6	32.6±9.1*	1550±474	2.10±0.21	26.9±8.5	1060±326*	2.53±0.07****	68.4±10	49.6±13.1	7.3	69.1±1.2
22A	73.1±28.0	22.4±6.6	36.0±11.3	34.0±10.7	1580±549	2.15±0.23	27.9±9.5	1120±381	2.49±0.06	71.2±5.3	50.0±8.2	6.9	69.0±2.3
23e	70.2±12.7	21.4±4.8	37.3±6.9	35.5±6.6	1610±325	2.20±0.14	30.1±5.9	1200±236	2.51±0.06**	74.4±6.1	56.0±7.9*	7.4	70.4±0.9

The data are presented as mean ± standard deviation, and analyzed with one-way ANOVA and Dunnett’s test. Statistical significance was indicated with

* (*p* < 0.05),

** (*p* < 0.01),

*** (*p* < 0.001),

**** (*p* < 0.0001).

### *Methylobacterium* species isolated from leaves of inoculated rice seed

The leaves of rice plants harbored approximately 0.7 to 1.4 × 10^5^ CFU of pink-pigmented *Methylobacterium* species per gram fresh weight irrespective of inoculation, the number of which was not affected by inoculation (data not shown). Roots were also sampled but no pink-pigmented colony was obtained from all samples (detection limit, 100 CFU/g fresh weight). These results suggested that leaves are the preferred niche for pink-pigmented *Methylobacterium* species compared with roots, as previously shown by metaproteogenomic analysis [30].

**Fig 1:**
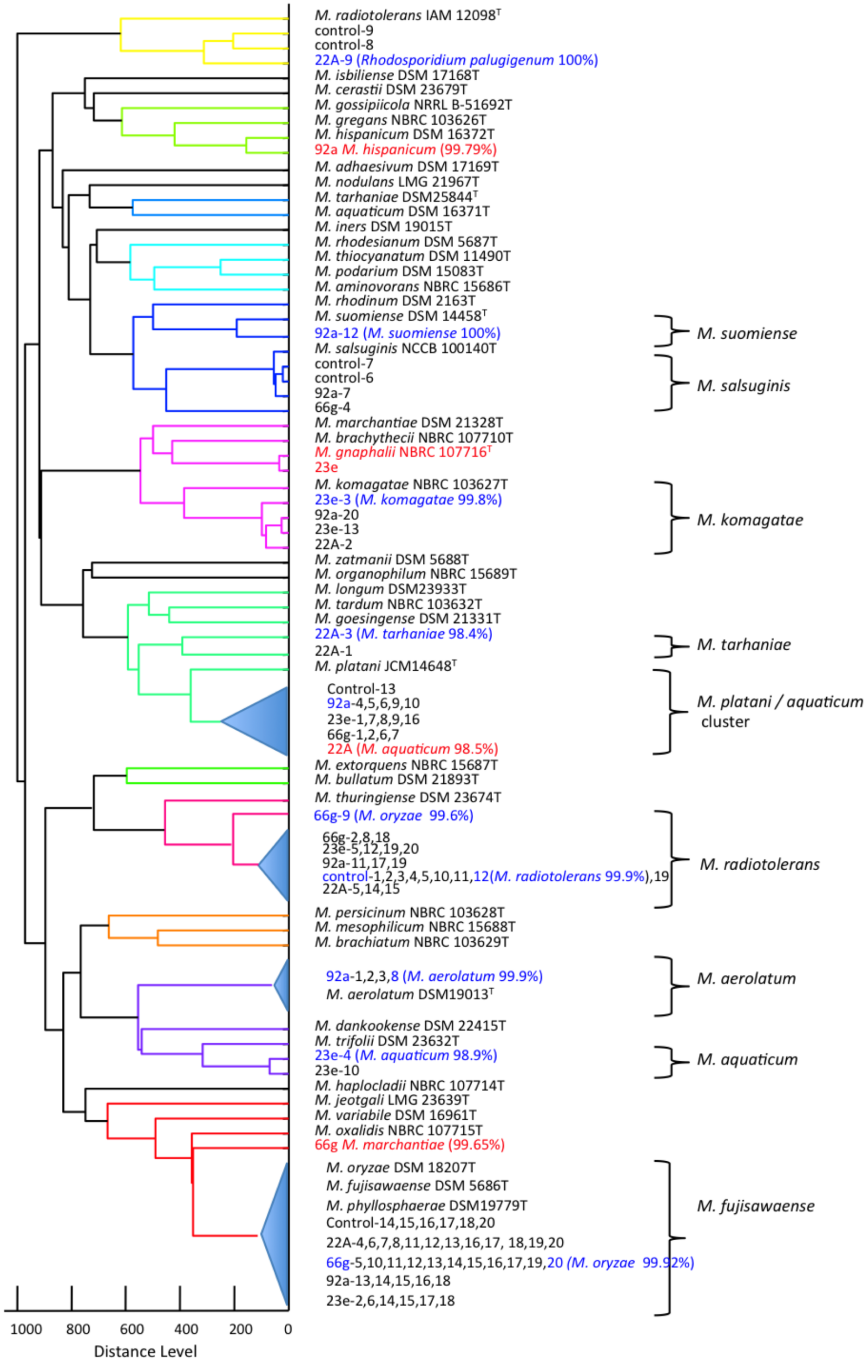
MSP dendrogram based on WC-MS analysis of the isolates from leaves of inoculated rice seed. The isolates were named with the treated strain name and isolate numbers. The inoculated strains are colored in red. The representatives selected from each cluster are colored in blue with the closest type strain name and percentage identity of 16S RNA gene in parentheses. Isolate 22A-9 was pink-pigmented fungus and was identified by ITS region sequencing, as described previously [19].

The WC-MS clustering result of the isolates from the cultivar Nipponbare, which had been subjected to seed-inoculation, is shown in Fig 1. In the default BioTyper settings clusters below distance level 700 are colored to indicate possible identity at species level. However, as shown in many combinations of the type strains (which are different at the species level), their distance level is sometimes lower than 700. Thus, we performed 16S rRNA gene sequencing for isolates located at branches of distance level 700–300; 98.6% identity was taken as the species threshold. Isolates sharing a distance level below 300 were regarded as the same species. The position of *M*. *hispanicum* strain 92a in the MSP dendrogram is also close to *M*. *hispanicum* type strain. The positions of *M*. *gnaphalii* strain 23e^T^ [26] and *M*. *marchantiae* strain 66g in the dendrogram are unique.

**Fig 2:**
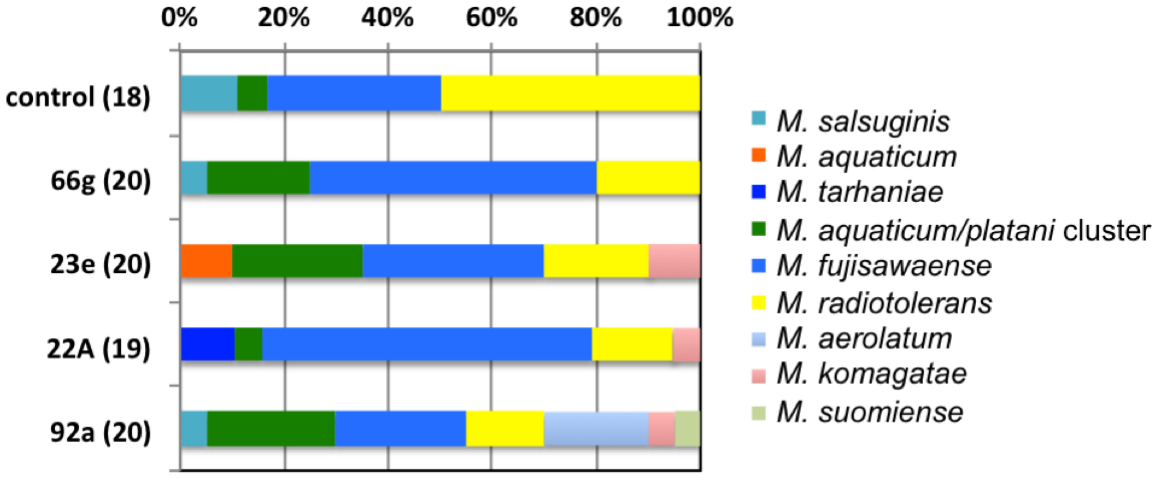
Identification and composition of *Methylobacterium* species isolated from rice plants subjected to *Methylobacterium* inoculation. Total number of *Methylobacterium* isolates is shown in parentheses. Identification was performed by WC-MS and 16S RNA sequencing, and details are shown in Fig 1.

Although we expected that the inoculated isolates could be recovered from the plants, the WC-MS identification of the isolates did not match the corresponding inoculants at all, suggesting that they had been eliminated from the plants during their growth in the field or unrecovered. Based on the MSP identity, the composition of *Methylobacterium* species in each sample is summarized in Fig 2. The un-inoculated control plants harbored isolates belonging to *M*. *salsuginis*, *M*. *aquaticum/platani* cluster, *M*. *fujisawaense*, and *M*. *radiotolerans*. With the exception of *M*. *salsuginis*, they were also detected from the inoculated samples as major species. The isolates from 66g-inoculated plants showed similar composition. 23e, 22A, and 92a-inoculated plants harbored several *M*. *aquaticum*, *M*. *tarhaniae*, and *M*. *aerolatum* and *M*. *suomiense*, respectively, as unique isolates. These unique isolates could only be isolated from the inoculated plants although they are not identical to the species of the inoculated isolates, suggesting that the inoculation influenced the *Methylobacterium* composition in the plants. This conclusion was supported by chi-squared test (*p* = 0.003) although the sample size (18–20 isolates from each) was small. The effect of inoculation that resulted in increased ripening might not be due solely to the change in *Methylobacterium* community, but also to changes in communities of other bacteria.

Isolates belonging to *M*. *radiotolerans* and *M*. *fujisawaense* were obtained from rice seedlings of cultivar Kinuhikari in a previous study [31], and those belonging to *M*. *mesophilicum* in addition to *M*. *radiotolerans* were from cultivar Koshihikari [32], using nutrient agar medium. No isolate belonging to *M*. *mesophilicum* was obtained in the current study, which was probably in part because of the different media used for isolation or the different rice preparation.

### Growth promotion of barley by *Methylobacterium* species

Among the 77 isolates in our library that were tested, 19 were selected through the first screening (S1 Table). Due to high variation in the barley growth, the second screening did not result in a reproducible growth promotion effect; however, the six most effective isolates were selected based on these screening processes (*M*. *extorquens* strain 21C, *M*. *brachiatum* strain 66e, *M*. *komagatae* strain z61e, *M*. *thiocyanatum* strain 87a, *M*. *aerolatum* strain z18b, and *M*. *variabile* strain 90a). The result of pot cultivation using these isolates is summarized in Table 2. During the growth, the height of inoculated plants never exceeded that of non-inoculated plants (data not shown), and inoculated plants tended to show smaller height and dry mass weight. A marked decrease in grain number and weight was observed in 66e-inoculated plants. Except for z18b, inoculation resulted in a decreased grain number compared to the control. Together, these results indicated increased grain size, which was evident in seed thickness. Thus, inoculation of *Methylobacterium* species resulted in fewer grains of greater size, and did not result in increased yield. The protein content tended to decrease in inoculated plants, whereas the starch content was not affected. The increase in grain size is of interest when considering the practical application for barley production, since small grains are eliminated at quality control. Field-level tests should be conducted to evaluate the applicability.

**Table 2:** Growth of *Methylobacterium*-inoculated barley.

Inoculated isolates	Plant height (cm)	Dry mass weight (g)	Grain (no. per plant)	Grain weight (g)	100-grain weight (g)	Seed thickness (mm)	Protein content (%)	Starch content (%)
control	81.3±5.1	13.3±2.1	267±40	6.72±1.27	2.52±0.26	2.29±0.2	8.7	60.7
21C	71.5±17.5*	13.3±2.4	249±48	7.18±1.62	2.89±0.22	2.4±0.23**	9.0	61.1
66e	71.3±7.3*	9.7±1.3***	181±19****	5.15±0.58**	2.84±0.17**	2.4±0.21**	7.5	58.7
z61e	72.7±6.8	12.3±2	217±39*	6.26±1.17	2.89±0.27	2.46±0.22**	6.9	59.4
87a	71.5±7.1*	11.1±2.4	199±42***	5.92±1.1	2.97±0.3	2.49±0.16****	6.8	60.1
z18b	78.3±5.8	14.4±4	280±81	7.27±2.05	2.63±0.31	2.32±0.28	7.2	61.2
90a	71.6±10.7*	11.8±2.3	234±43	6.02±1.19	2.58±0.34	2.31±0.24	8.5	62.0

The data are presented as mean ± standard deviation, and analyzed with one-way ANOVA and Dunnett’s test. Statistical significance was indicated with

* (*p* < 0.05),

** (*p* < 0.01),

*** (*p* < 0.001),

**** (*p* < 0.0001).

### Isolation and identification of *Methylobacterium* species from various cultivars of barley

**Fig 3:**
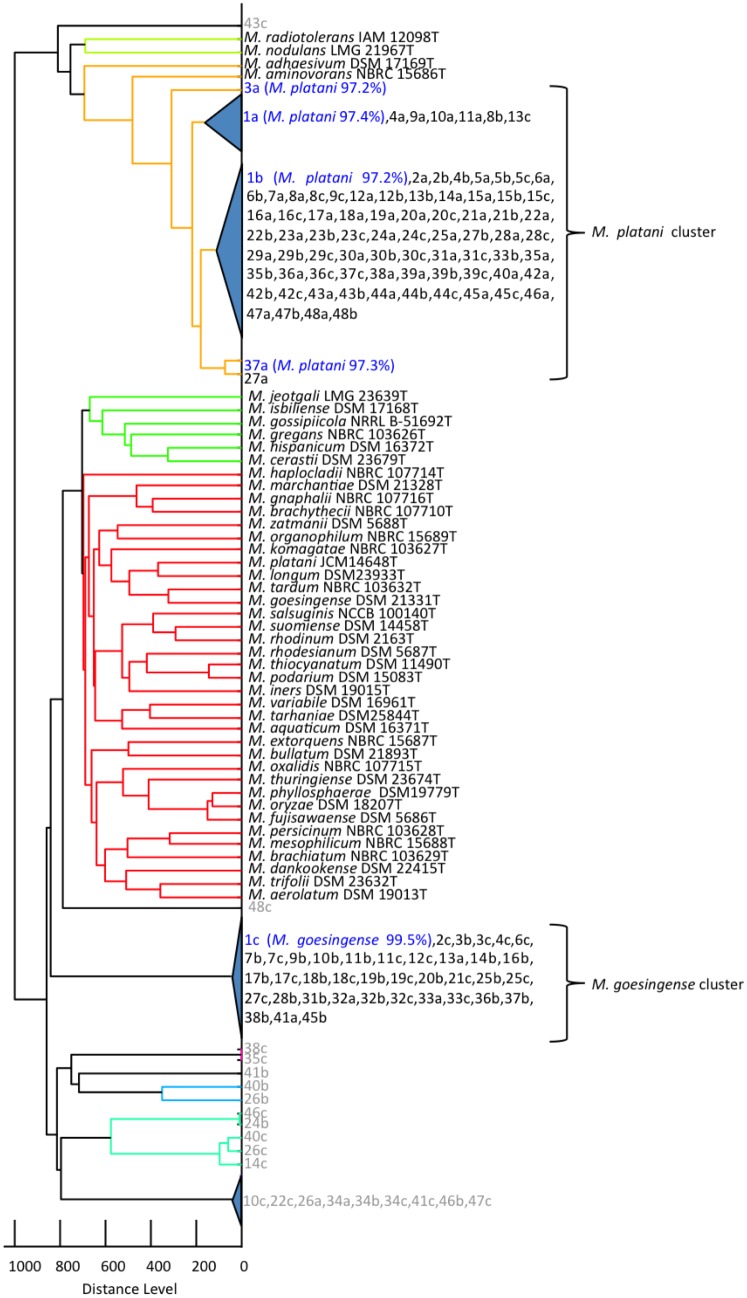
MSP dendrogram based on WC-MS analysis of the isolates from barley of different cultivars. The representatives are shown in blue with the closest species name and percentage identity of the 16S RNA gene in parentheses. Strains shown in gray were not pink-pigmented.

Fig 3 shows an MSP dendrogram of the isolates from various barley cultivars and one wheat cultivar. Three isolates from each cultivar were coded together with sample numbers (S2 Table). Based on the WC-MS clustering result, the isolates from cultivars could be grouped into two clusters, *M*. *platani* and *M*. *goesingense*. The low (<97.5%) pairwise nucleotide identity of the *M*. *platani* cluster necessitates further taxonomic studies. The isolates positioned out of these two clusters were non-pigmented methylotrophs, and they do not belong to the genus *Methylobacterium* (data not shown).

Overall, *Methylobacterium* isolates from various cultivars belong to only two of these species, indicating the narrow interaction specificity between them. Fisher’s two-sided exact test showed a slightly significant difference in the isolate genotypes (*M*. *platani*, *M*. *goesingense*, and the others) between malting and non-malting barley (*p* = 0.12) (Table 3). Kernel row type affected the isolate genotypes significantly (*p* = 0.0019). These results suggested that malting or two-rowed barleys contained more isolates of *M*. *goesingense* than those of *M*. *platani*. Two-rowed barleys are mainly used for malting, whereas six-rowed barleys are used for foods and feeds. Malting barleys require specific quality profiles of 60–65% starch and 10.5–13.5% protein [33]. Uniform and fast germination is also necessary in malting barleys to activate enzymes for brewing. These biochemical requirements are specific to malting barleys and may affect the host preference of *Methylobacterium* species. Interestingly, *M*. *goesingense* has not been isolated from the rice grown in the same field in different seasons.

**Table 3:** Isolates’ genotypes and characteristics of barley.

	Isolated species			
Barley characteristics	*M*. *platani*	*M*. *goesingense*	Others	Total
Malting (12)	18 (52.9%)	15 (44.1%)	3 (8.8%)	36 (100%)
Non-malting (21)	37 (66.1%)	13 (23.2%)	13 (23.2%)	63 (100%)
Two-row (26)	40 (51.3%)	24 (30.8%)	14 (17.9%)	78 (100%)
Six-row (21)	43 (68.3%)	13 (20.6%)	7 (11.1%)	63 (100%)

### Phylogenetic analysis of *Methylobacterium* isolates from rice and barley

**Fig 4:**
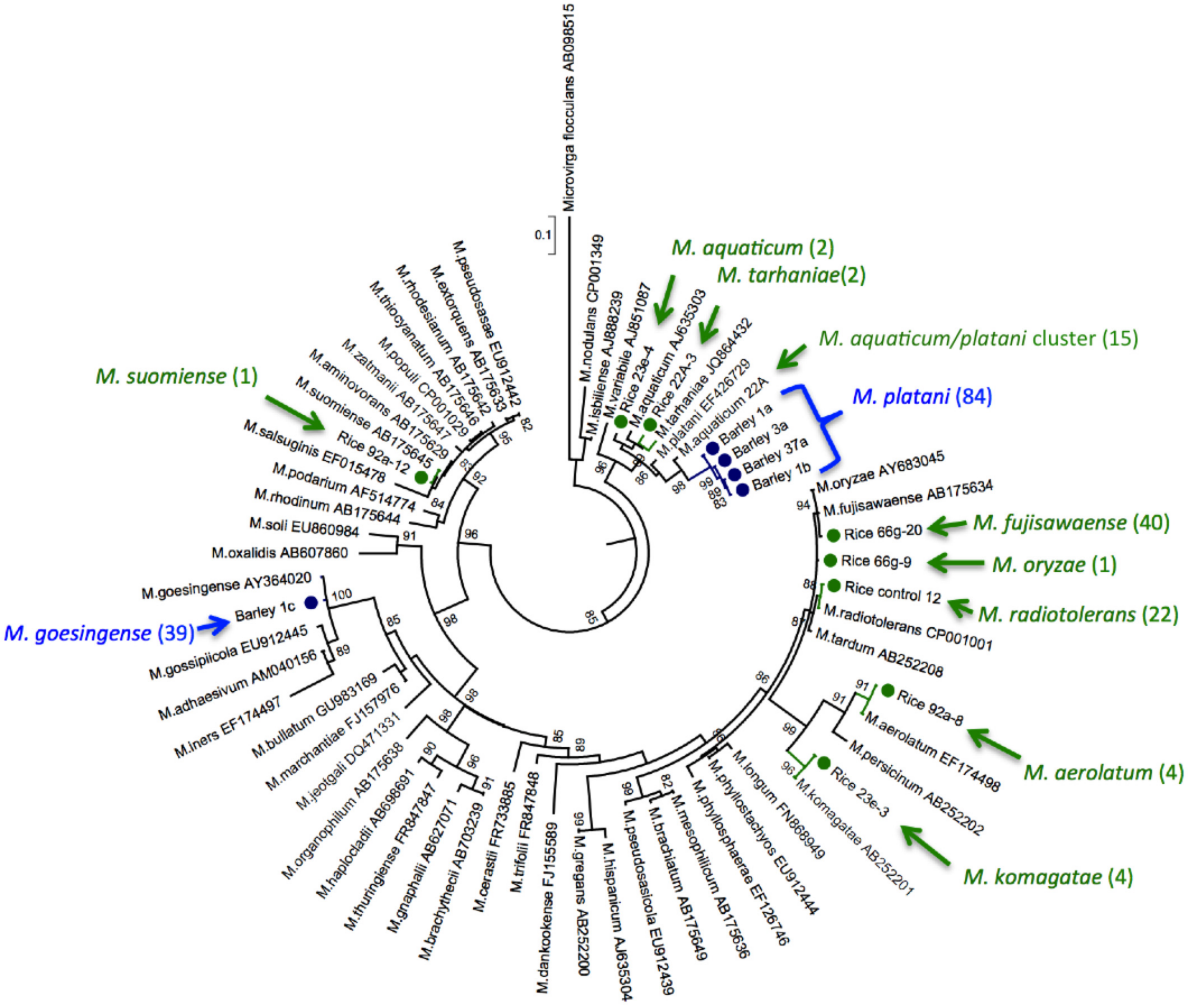
Maximum-likelihood phylogenetic tree of *Methylobacterium* isolates and related taxa, based on 16S rRNA gene sequences. Isolates from rice are colored in green and those from barley are in blue. Numbers in parentheses indicate isolates belonging to the same species, estimated by WC-MS analysis. For isolates from rice, the inoculation effect is not taken into account in the figure. *M aquaticum* strain 22A is taken as a representative strain for the *M*. *platani/aquaticum* cluster shown in Fig 1. Bootstrap percentages based on 1000 replicates are shown if greater than 80%. *Microvirga flocculans* TFB (AB098515) was used as an outgroup. Bar, 0.1 changes per nucleotide position.

The maximum-likelihood phylogenetic tree of *Methylobacterium* isolates was constructed and is shown in Fig 4. As indicated in Figs 1–3, many of the isolates from rice belong to the species of *M*. *fujisawaense*, *M*. *radiotolerans*, and *M*. *platani/aquaticum*, and those from barley and wheat belong to *M*. *goesingense* and *M*. *platani* group. The analysis indicated that the isolates from rice and those from barley and wheat belong to completely different species, although they were grown in the same field in different seasons. Although they belong to the same plant family, *Poaceae*, there were many differences in environmental factors that might have affected the *Methylobacterium* species composition. Considering the wide variety of species in the genus *Methylobacterium*, the interaction specificity of them associating with rice and especially with barley found in this study is strikingly narrow, and the mechanism of this remains unknown at the moment.

## Conclusions

In conclusion, we screened *Methylobacterium* isolates from our laboratory collection for high rice and barley seedling growth promotion, and then tested them in field and pot conditions. Even though the tested strains showed a growth promotion effect *in vitro*, what we observed in the field and pot experiments was not apparent growth promotion, but increased quality of grains. It should be investigated whether the involvement of cytokinin synthesis in *Methylobacterium* species is the cause. In the case of rice, it might not be a direct effect of the inoculated strains, since they could not be recovered from the inoculated rice. However, the inoculation might affect the community structure that might further affect plant growth. We suggest that, in order to achieve a positive effect of growth promotion by *Methylobacterium*, a continuous application of cells would be necessary to ensure colonization of the inoculated strains. It is true that *Methylobacterium* species reside in naturally grown healthy plants and that they are one of the major bacteria residing in the phyllosphere. Thus, it may be more important to use the isolates obtained from the plant of interest, since they must have fitness to the plant and the environment. At the same time, the predomination of such limited species occurring even after artificial inoculation suggests that there is a species-level selection pressure that is strong enough for the coming species to outcompete the inoculated species. Recently, it was reported that biofilm formation is enhanced when two different *Methylobacterium* strains were co-cultured [34]. Thus, it is also possible that a mixture of specific different species works together to inhabit a specific plant surface. Finally, although it is restricted to isolated strains, the WC-MS technique used in this study is applicable not only to the rapid identification and classification of unidentified isolates, but also to the estimation of the variety and relative abundance of species from different samples.

## Supporting Information

S1 Data WC-MS spectra data of *Methylobacterium* type strains. (ZIP)

S2 Data WC-MS spectra data of *Methylobacterium* isolates from inoculated rice. (ZIP)

S3 Data WC-MS spectra data of *Methylobacterium* isolates from barley. (ZIP)

S1 Table Barley cultivars used for *Methylobacterium* isolation. (XLSX)

S2 Table The isolates used for rice and barley growth promotion screening and the results. (XLSX)
